# Simple, efficient and economical methods for isolation and estimation of novel isoflavone using RP-HPLC

**DOI:** 10.1016/j.mex.2017.02.001

**Published:** 2017-02-23

**Authors:** Afroze Alam, Kamlesh Kumar Naik, Navneet Kumar Upadhaya, Shailendra Kumar, K.L. Dhar

**Affiliations:** a School of Pharmaceutical Sciences, Shoolini University, Bajhol, Solan, 173229, Himachal Pradesh, India; b Nandha College of Pharmacy, Perundurai Main Road, Erode, Tamil Nadu, 638052, India; c Government Pharmacy Institute, Agam Kuan, Patna, 800007, Bihar, India

**Keywords:** *Iris kashmeriana*, Isoflavone, RP-HPLC, Spectroscopic chracterization, Quantitative estimation

## Abstract

The study was undertaken to develop a simplified procedure for the isolation of bioactive isoflavone from *Iris kashmiriana*, using a direct method of isolation, avoiding the use of chromatographic techniques. The compound was isolated by commercially viable procedure. The extraction of powdered drug (500 g) was done with petroleum ether (60–80) using a Soxhlet apparatus (24 h run). The petroleum ether extract (gums and resins 2.13 g) was obtained and the marc (400 g) was subjected to extraction with 95% methanol using a Soxhlet apparatus (24 h run). The methanolic extract (5 g) was subjected to successive fractionation with toluene, chloroform and ethyl acetate and n- butanol. On the basis of phytochemical analysis, the glycoside was present in n- butanol fraction. The *n*-butanol fraction (1.5 g) was taken in dried methanol, passed through activated animal charcoal and subjected to acid hydrolysis. The isoflavone (250 mg), was obtained after the usual process of separation. The purity of the compound was checked by analyzing TLC (Thin Layer chromatography) and melting point. Further, the chemical method was used to characterize the compound by shift reagents using UV spectroscopy. The quantitative estimation of isoflavone was done using RP-HPLC and was found to be 98.9% pure.

• The “previously undescribed” isoflavone was isolated by modifying approach of solvent/solvent extraction, fractionation and acid hydrolysis.
• The spectroscopic characterization was equaly done by IR, ^1^HNMR, ^13^CNMR, Mass spectrometry.
• 98.9% purity was achieved using RP-HPLC with simple solvent (Methanol and Water 55: 45).

## Graphical abstract

**Figure:**
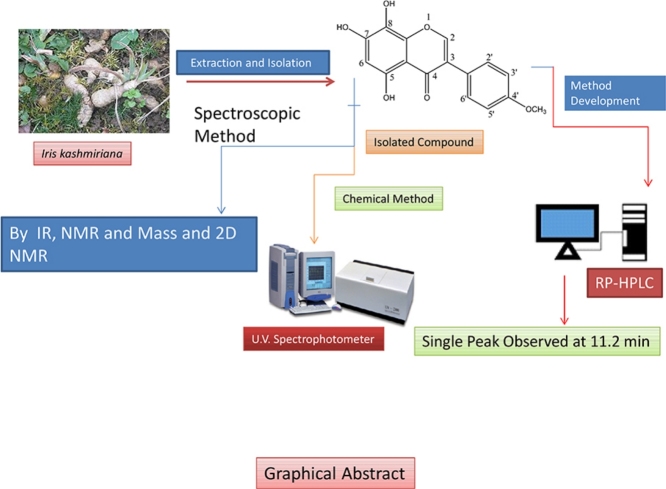

## Method details

### Extraction and isolation

Solvent-solvent extraction technique was adopted for the isolation of novel compound. In continuing with this technique the rhizomes of *Iris kashmiriana* Baker were dried, chopped, and powdered (500 g). The extraction of powdered drug was done with petroleum ether (60–80) using a Soxhlet apparatus (24 h run). The petroleum ether extract (gums and resins 2.13 g) was obtained and the marc (400 g) was subjected to extraction with 95% methanol using a Soxhlet apparatus (24 h run). The methanolic extract (5 g) was subjected to successive fractionation with toluene, chloroform and ethyl acetate and n- butanol [1]. On the basis of phytochemical screening results, the glycoside was present in *n*- butanol fraction. The *n*-butanol fraction (1.5 g) was taken in dried methanol (80%), passed through activated animal charcoal. The acid hydrolysis was carried out in the presence of 5% HCl (the reaction mixture was refluxed for 2–3 h at 45–50 °C and kept for night) [2]. Completion of reaction was monitored on TLC (10% Methanol in Toluene). The solution was poured into water and the mixture was separated out by separating funnel. From organic layer, the solvent was removed by distillation and residue was further dried. The identification of compound was confirmed by TLC in chloroform: methanol 9:1 with Rf value 0.65 [3]. The structure of the compound was characterized and established by various spectroscopic techniques viz, UV, IR, ^1^HNMR, ^13^CNMR, Mass spectrometry and 2D NMR. Furthermore, the compound was also characterized by chemical methods by shift reagents (AlCl_3_ and CH_3_COONa) using UV spectrophotometer [4]. Finally, the compound was considered as an aglycone/isoflavone (250 mg). The aqueous portion of the above was subjected to paper chromatography [5] using *n-*butanol, acetone and water in the ratio 4:1:5, where it was clearly observed that the sugar attached was glucose, with Rf value 0.68.

Why this method was adopted in the present work?

• This method was simple, modified, reproducible, efficient, eco-friendly and cost effective. Thus, the present work avoided the use of hyphenated chromatographic techniques such as Column chromatography & Preparative TLC (Thin Layer Chromatography).
• The study deals with the isolation of “previously undescribed” molecule, together with the identification of sugar moiety from the glycoside.

## Quantitative estimation of isoflavone by RP-HPLC

### Sample preparation

10 mg test sample was dissolved in 100 mL of methanol to prepare 100 ppm solution; further dilution was made to prepare 10 ppm solution.

### Protocol for RP-HPLC

**Fig. 1:**
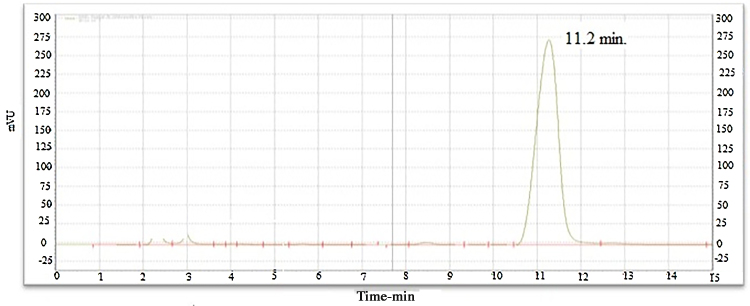
Chromatogram of new isolated isoflavone showing single peak.

A simple, sensitive and accurate RP- high performance liquid chromatography (RP-HPLC) method has been used for the quantitative estimation [6], [7] of “previously undescribed” isoflavone from *Iris kashmiriana* Baker. Agilent 1200 series HPLC binary pump system was performed on a C18 column with water: methanol (45:55), as mobile phase. Detection was performed by the DAD detector at 338 nm. This technique has been applied, for the quantitative estimation of new isoflavone. The retention time of the isoflavone was 11.2 min and no other peaks were observed. Injection volume was taken 10 μL. The purity of the compound was found to be 98.9% with single peak, which indicates the compound isolated was substantially pure and single; moreover the present study appears to be first of its kind of approach (Fig. 1).

What is the significance adopted in the present work?

• Quantitative estimation of isoflavone was done by RP-HPLC using mobile phase (water: methanol; 45:55) and the method was developed without using costly solvents such as acetonitrile. The single peak was observer after 11.2 min with significant purity (98.9%).

## Details methodology of UV spectroscopy analysis

Ultraviolet and visible spectroscopy was one of the basic techniques routinely used for flavonoid/isoflavanoid analysis due to the existence of two characteristic UV/Vis bands in flavonoids, band I in the 300–550 nm range, arising from the B ring, and band II in the 240–285 nm range, arising from the A ring, with some variations.

Shift reagents, such as aluminium chrolide and sodium acetate, lead to shifts in the maximum wavelength of these bands due to acetate-induced deprotonation of OH groups or Al^3+^ complexation by OH groups, were also used to study flavonoid/isoflavanoid structure.

## Chemical methods of characterization of isoflavone by U.V. Spectrophotometer [8]

### Aluminium chloride (AlCl_3_ shift)

The test compound (10 mg) was dissolved in 100 mL methanol and λmax was determined (341 and 238.4 nm). When few drops of AlCl_3_ was added, a complex was formed between carbonyl group, hydroxyl group (at C-5) and AlCl_3_, resulting in the shift of λmax from 341 nm to 377.6 nm and 238.4 nm to 264.8 nm, respectively. In continuation with the procedure, few drops of HCl was added in the same mixture, there was no change in the shift of λmax except the optical density. This clearly indicates the vicinity of hydroxyl groups at C5- OH and carbonyl (>C=O at C4) in the isoflavone moiety (Table 1, Table 2).

**Table 1:** Chemical characterization of compound by UV Spectrophotometer using AlCl_3_.

	Sample + solvent	λmax^a^	Absorbance
1	Comp + Methanol	341 nm, 238.4 nm	1.257 and 0.990
2	Comp + Methanol + AlCl_3_	377.6 nm, 264.8 nm	0.291and 1.217
3	Comp + Methanol + AlCl_3_ + HCl	377.6 nm, 264.8 nm	0.328 and 1.381

a λmax was determined according to the Lambert’s & Beer Law.

**Table 2:** Chemical characterization of compound by UV Spectrophotometer using CH_3_COONa.

Sl. no	Sample + solvent	λmax^a^	Absorbance
1	Comp + Methanol	341 nm, 238.4 nm	1.257 and 0.990
2	Comp + Methanol + NaOCOCH_3_	341 nm, 238.4 nm	0.7250 and 1.084

a λmax was determined according the Lambert’s &Beer Law.

### Sodium acetate (CH_3_COONa) Shift

The compound (10 mg) was dissolved in 100 mL methanol and λmax was determined.

Since, no shift in λmax has been observed in UV region (341 nm, 238.4 nm) after the addition of sodium acetate, thereby it clearly indicated, that the absence of free hydroxyl groups at C-3′,C-5′ and C-4′ positions.

Why this method was adopted in the present work?

This technique is inexpensive, reproducible and less time-consuming to study the flavonoids/isoflavonoid structure, though the other methods of characterization are equally important. Nowadays these techniques are not routinely used but still continue to be applied in some cases, in particular to HPLC eluates – a hyphenated LC-UV-MS has been developed using post-column UV shift reagents for the flavonoid/isoflavonoid analysis of crude extracts.

### Other methods of characterization

**Fig. 2:**
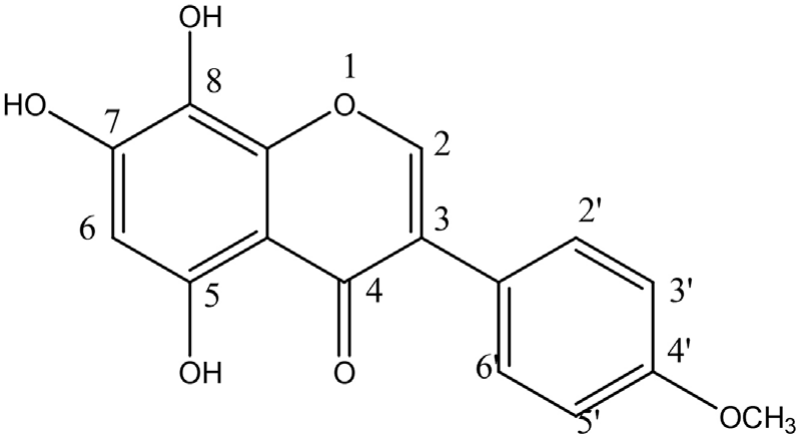
5,7,8-trihydroxy-3-(4-methoxyphenyl)-4-one.

The aglycone was isolated, purified and recrystallized with methanol, the melting points was found to be 225–227 °C (uncorrected) and further analyzed for C_14_H_12_O_6_. The elemental analysis found (%) C;64.00,H;4.03,O;31.97 and calculated; C;63.21,H;3.40, O;30.83. Mass spectrometry analysis by TOF,MS, ES+. (m/z) = 301.26.The structure was further assigned by IR,^1^H NMR, C^13^ NMR, Mass spectrometry, together with U.V. RP- HPLC and chemical methods [5]. The structure of isolated isoflavone is given below (Fig. 2).

Spectroscopic characterization

• IR(KBr, cm^−1^): 1518 (ArC=C)], 1659 (C=O), 3080 (ArC-H), 1372 (C-O), 2825(C-H Str), 3368 (Ar —OH).
  The IR data indicates the presence of carbonyl group, aromatic ring and hydroxyl groups.
• ^1^H-NMR(400 MHz, δ, DMSO, TMS = 0): 3.89 (3H,s, 4′-H), 6.46 (1H,s,6-H) 6.85 (2H, d,3′,5′-H, *J* = 8.60 Hz), 7.37 (2H,d, 2′,6′-H,*J* = 8.64 Hz), 8.15(1H,s,2-H),
• The structure was further confirmed by ^13^C- NMR assignment.
  ^13^C- NMR (400 MHz, δ, DMSO, TMS = 0): C-4, 180.50, C-4′ 157.39, C-5 153.22 C-7 153.30, C-2 153.31, C-6 131.22, C-8a 121.92, C-8 132.47, C-3′,5′, 115.01, C-2′,6′ 129.91, C-1′,104.89, C-3 121.14, C-4a 93.71,CH_3_-59.80
• Mass spectrometry
  TOF MS ES^+^ 2.33e.3 m/z (rel. int): 301[M]^+^ (45.94), 294[M-6H]^+2^(13.12),285[M-CH_3_] + (36.79),269[M-CH_3_-O] + (12.05),253[M-CH_3_-O-O] + (63.63),237 [M-CH_3_-O-O-O] + (10.32), 208 [M-CH_3_-O-O-CO_2_] + (7.01),201 [M-C_6_H_4_CO ring B] + (6.63), Ring B = m/z 100. 187[M-CH_3_-O-O-O-C_4_H_2_] + (61.82),178[M-C_6_H_4_CO—C=C—] + (28.26),162[M-C_6_H_4_CO—C=C-O] + (34.94),146[M- C_6_H_4_CO—C=C-O-O-] + (22.05), 136[M- C_6_H_4_CO—C=C-O-C_2_H_2_] + (7.05), 117[M-CH_3_-3O-ring A] (4.91). Ring A = m/z116. 107[M- C_6_H_4_CO—C=C-O-C_2_H_2_-CHO](59.13). 85[117-2O](100)

## Additional information

With the help of above methodology, our team isolated more than 10 new molecules from different plant species. For example, karanjin and their new analogues are isolated from seed oils of *Pongamia pinnata*, Lactone and their analogues are isolated from *Inula resemosa* (Pushkarmool).The RP-HPLC method developed can also be extrapolated for the standardization of herbal formulation containing isoflavone.
